# Supplementary material for: Loneliness corresponds with neural representations and language use that deviate from shared cultural perceptions
Source: Commun Psychol. 2024 May 6;2:40. doi: 10.1038/s44271-024-00088-3 (PMC11073992; doi:10.1038/s44271-024-00088-3)
Supplement: Supplementary file 2 — Supplementary Information [file 44271_2024_88_MOESM2_ESM.pdf]

SUPPLEMENTARY INFORMATION:

Loneliness corresponds with neural representations and language use that deviate from shared cultural perceptions

Timothy W. Broom[1], Siddhant Iyer[2], Andrea L. Courtney[3], Meghan L. Meyer[1*]

1. Department of Psychology, Columbia University, New York, NY, USA
2. Department of Neuroscience, Columbia University, New York, NY, USA
3. Department of Psychology, Stanford University, Stanford, CA, USA

[*]Corresponding author:

Meghan L. Meyer: mlm2378@columbia.edu

Supplementary Results

Simulations exploring the independence of results focused on pairwise similarity versus similarity to the group-consensus. In the main text, we outline the conceptual differences between the analyses focused on similarity between pairs of participants in their representations of celebrities versus the similarity of each individual participant's representation of a celebrity to the group-consensus representation. Here we additionally simulate data in order to demonstrate that these effects are not mutually inclusive, i.e., that one can be statistically significant while the other is not statistically significant and vice versa. We randomly generated X and Y coordinates (bound between zero and one) for 21 "low-loneliness" participants (a median split on loneliness scores in Study 1A resulted in uneven group sizes due to some participants having the same loneliness scores), and 19 "high-loneliness" participants. The randomly generated coordinates for the "high-loneliness" participants were then multiplied by 1.5 to create greater distance between these points in line with the hypothesized pairwise effect (i.e., greater dissimilarity among lonelier pairs in their representations of celebrities). We then examined how the results of both the analysis focused on pairwise similarity (i.e., its association with pairs' mean loneliness scores) and that focused on similarity to the group-consensus (i.e., its association with loneliness scores) changed depending on where the "high-loneliness" cluster of participants was located relative to the "low-loneliness" cluster of participants. Figures S1-S3 depict three different patterns of results depending on where the two clusters are relative to one another: a significant association for pairwise similarity and non-significant association for similarity to the group-consensus (Figure S1), a non-significant association for pairwise similarity and significant association for similarity to the group-consensus (Figure S2), and a significant association for both (Figure S3). These first two examples illustrate the point that it is

possible for these two patterns of results to exist independent of one another and that, though related, they are not redundant with one another.

Study 1.

Reliability-based voxel selection. All analyses were focused on two regions of interest (ROI) defined using reliability-based voxel selection[1] (RBVS), one in the medial prefrontal cortex (MPFC) and one in the precuneus (PC)/posterior cingulate cortex (PCC). On page 13, we include Fig. S4, which includes plots depicting how the ROIs were defined through RBVS as well as plots of the resulting ROIs in the MPFC and PC/PCC.

Results excluding participants "not at all" familiar with target celebrities. As described in the materials and methods section of the main text, multiple participants in Study 1 provided familiarity ratings of 0 on a scale ranging from 0 (*not at all*) to 100 (*very much*) for some of the target celebrities. It is unclear based on these ratings alone whether being "not at all" familiar with a celebrity indicates that that participant had never heard of that person, or, though they had heard of that person before, that they subjectively felt they knew virtually nothing about them relative to their friends and acquaintances for whom they were also providing these ratings. Because the former remains a possibility, we conducted additional analyses to ensure that all results held when these observations were excluded. In Study 1A, one participant reported being "not at all" familiar with Justin Bieber and one reported being "not at all" familiar with Kim Kardashian. The results reported in the main text held when excluding these two observations. Specifically, pairs of participants with greater mean loneliness scores were more dissimilar in their neural representations of celebrities in the MPFC (β = -0.14, SE = 0.05, $t_{(39)}$ = 2.91, p = .003, 95% CI = -0.25 to -0.04), and this association was moderated by level of consensus as indicated by a significant interaction between mean loneliness scores and the contrast of high vs.

low consensus (β = -0.03, SE = 0.01, $t_{(3780)}$ = 2.05, p = .04, 95% CI = -0.05 to -0.0004). There was also evidence of moderation by celebrity type as indicated by a significant interaction between mean loneliness scores and the contrast of pop culture celebrity vs. political/business celebrity (β = -0.03, SE = 0.01, $t_{(3780)}$ = 3.20, p = .001, 95% CI = -0.05 to -0.01). In addition, lonelier individuals were more dissimilar to the group-consensus neural representations of celebrities in the MPFC (β = -0.27, SE = 0.13, $t_{(38)}$ = 2.04, p = .02, 95% CI = -0.54 to -0.03).

In Study 1B, four participants reported being “not at all” familiar with Justin Bieber, five reported being “not at all” familiar with Ellen DeGeneres, and four reported being “not at all” familiar with Kim Kardashian. The results reported in the main text held when excluding these 13 observations. Specifically, pairs of participants with greater mean loneliness scores were more dissimilar in their neural representations of celebrities in the MPFC (β = -0.17, SE = 0.07, $t_{(36)}$ = 2.57, p = .007, 95% CI = -0.31 to -0.03). In addition, lonelier individuals were more dissimilar to the group consensus neural representations of celebrities in the MPFC (β = -0.30, SE = 0.15, $t_{(35)}$ = 1.94, p = .03, 95% CI = -0.58 to -0.02).

Study 2. In the main text we report results demonstrating that for high-consensus celebrities, pairs with greater mean loneliness were lower in pairwise semantic similarity in their paragraphs describing those celebrities. In other words, lonelier individuals were more idiosyncratic in the meaning of the paragraphs they wrote about prominent pop culture celebrities. Here we report the results of control analyses conducted to determine what other variables explained semantic similarity in pairs’ descriptions of high-consensus celebrities, and, more importantly, whether associations with loneliness remained significant after controlling for these other variables. As can be seen in Supplementary Table 2, a number of other variables predicted pairwise semantic similarity in descriptions of high-consensus celebrities including

similarity in age, similarity in ratings of liking, similarity in perceptions of their traits, and similarity in number of words written. However, as can be seen in Supplementary Table 3, when these other predictors were entered simultaneously into a model predicting pairwise semantic similarity along with pairs' mean loneliness scores, mean loneliness remained a significant predictor (β = -0.06, SE = 0.03, $t_{(445)}$ = 2.27, p = .01, 95% CI = -0.12 to -0.008). This result indicates that the finding that lonelier pairs were more idiosyncratic in the semantic meaning of their descriptions of celebrities was independent of any shared variance with similarity in age, gender, perceptions of traits, word count, or subjective psychological closeness.

As can be seen in Supplementary Table 4, with respect to similarity to the group-consensus semantic representation of celebrities, race, similarity to group-consensus perceptions of celebrities' traits, and word count were associated with similarity to the group-consensus semantic representation of celebrities. However, when entering loneliness scores, race, similarity to group-consensus perceptions of celebrities' traits, and word count into a model predicting similarity to the group-consensus semantic representation of celebrities, loneliness remained a significant predictor (loneliness: β = -0.08, SE = 0.04, $t_{(441)}$ = 1.77, p = .04, 95% CI = -0.17 to -0.001; race: β = -0.19, SE = 0.10, $t_{(441)}$ = 1.78, p = .08, 95% CI = -0.40 to 0.02; similarity to group-consensus perceptions of traits: β = 0.15, SE = 0.05, $t_{(424)}$ = 3.40, p < .001, 95% CI = 0.07 to 0.24; word count: β = 0.28, SE = 0.04, $t_{(444)}$ = 6.34, p < .001, 95% CI = 0.20 to 0.37).

Finally, all analyses undertaken for semantic representations of celebrities were repeated for ratings of the celebrities' traits. A median split was used to divide the ten pop culture celebrities chosen for the study into two groups based on the mean pairwise trait perception similarity for each celebrity: high-consensus celebrities (Cameron Diaz, Harrison Ford, Michael Jordan, Keanu Reeves, and Justin Timberlake) and low-consensus celebrities (Justin Bieber,

Ellen DeGeneres, Megan Fox, Kim Kardashian, and Will Smith). There was no statistically significant interaction between mean loneliness and level of consensus, whether modeled as the contrast of high vs. low consensus celebrities (β = -0.03, SE = 0.02, $t_{(916)}$ = 1.60, p = .11, 95% CI = -0.07 to 0.003) or modeled continuously as each celebrity's mean pairwise trait perception similarity (β = -0.02, SE = 0.02, $t_{(915)}$ = 1.28, p = .20, 95% CI = -0.05 to 0.01). Further, there was no statistically significant association between mean loneliness and pairwise similarity in perceptions of celebrities' traits whether including data from all celebrities (β = 0.02, SE = 0.02, $t_{(913)}$ = 1.13, p = .26, 95% CI = -0.02 to 0.06), or just high-consensus celebrities (β = -0.01, SE = 0.03, $t_{(447)}$ = 0.38, p = .70, 95% CI = -0.06 to 0.04). There was also no statistically significant association between loneliness and similarity to the group-consensus perception of a celebrity's traits for high-consensus celebrities (β = -0.01, SE = 0.05, $t_{(448)}$ = 0.13, p = .90, 95% CI = -0.11 to 0.09).

Supplementary Table 1. *Independent Samples T-Tests Assessing Differences in Distributions of Pairwise Semantic Similarity for All Pairs of Celebrity Targets.*

**First Celebrity**	**Second Celebrity**	**Satterthwaite Approximated *df***	***t* Value**	***p* Value**
Kim	Ellen	179	1.21	.23
Harrison	Ellen	158	2.48	.01
Michael	Ellen	175	0.58	.56
Ellen	Keanu	180	0.79	.43
Ellen	Justin B.	192	1.11	.27
Ellen	Cameron	163	1.21	.23
Ellen	Megan	167	2.26	.03
Ellen	Will	174	1.74	.08
Ellen	Justin T.	184	2.81	.006
Harrison	Kim	170	1.11	.27
Kim	Michael	187	0.63	.53
Kim	Keanu	192	2.02	.04
Kim	Justin B.	205	2.51	.01
Kim	Cameron	175	2.37	.02
Kim	Megan	180	3.50	<.001
Kim	Will	186	3.06	.003
Kim	Justin T.	196	4.15	<.001
Harrison	Michael	166	1.79	.08
Harrison	Keanu	171	3.30	.001
Harrison	Justin B.	183	4.13	<.001
Harrison	Cameron	154	3.7	<.001
Harrison	Megan	158	5.22	<.001
Harrison	Will	165	4.88	<.001
Harrison	Justin T.	175	6.06	<.001
Michael	Keanu	188	1.37	.17
Michael	Justin B.	200	1.78	.08
Michael	Cameron	171	1.75	.08
Michael	Megan	175	2.84	.005
Michael	Will	182	2.36	.02
Michael	Justin T.	192	3.43	<.001
Keanu	Justin B.	205	0.21	.84
Keanu	Cameron	176	0.46	.65
Keanu	Megan	180	1.43	.15
Keanu	Will	187	0.85	.40
Keanu	Justin T.	197	1.90	.06
Justin B.	Cameron	188	0.33	.74
Justin B.	Megan	193	1.45	.15
Justin B.	Will	200	0.76	.45
Justin B.	Justin T.	210	1.98	.049
Cameron	Megan	163	0.90	.37

Cameron	Will	170	0.31	.76
Cameron	Justin T.	180	1.31	.19
Will	Megan	175	0.73	.46
Megan	Justin T.	185	0.37	.71
Will	Justin T.	191	1.19	.23

Note. Due to the non-independence of the data (i.e., participants were represented in multiple pairs), linear mixed-effects modeling was implemented to conduct the independent samples t-tests displayed in the table above with a random intercept for the first participant in a pair and the second participant in a pair. The observations for each test were doubled to allow each participant to be modeled as both the first and second participant in a pair for each observation (see methods section in main text). The resulting redundancy in the data was then accounted for by halving the estimated degrees of freedom and adjusting the p *value accordingly. Pairs who wrote about one celebrity were coded as 1.0 and pairs who wrote about the other celebrity were coded as 0.0. In the table above, the "First Celebrity" is always the one with higher mean pairwise semantic similarity. df = degrees of freedom. Ellen = Ellen DeGeneres. Kim = Kim Kardashian. Harrison = Harrison Ford. Michael = Michael Jordan. Keanu = Keanu Reeves. Justin B. = Justin Bieber. Cameron = Cameron Diaz. Megan = Megan Fox. Will = Will Smith. Justin T. = Justin Timberlake.*

Supplementary Table 2. *Single-Predictor Models Predicting Pairwise Semantic Similarity for Descriptions of High-Consensus Celebrities.*

**Predictor**	**Beta**	**Standard Error**	**Satterthwaite Approximated *df***	***t* Value**	***p* Value**	**95% CI**
Gender	0.02	0.01	19683	3.21	.001	0.01 to 0.03
Race	0.03	0.01	20101	3.43	<.001	0.02 to 0.05
Age	0.03	0.004	19928	7.62	<.001	0.02 to 0.03
Liking	0.04	0.003	19835	11.68	<.001	0.03 to 0.05
Similarity	0.01	0.003	19832	3.22	.001	0.005 to 0.02
Closeness	0.03	0.004	19897	8.58	<.001	0.02 to 0.04
Familiarity	0.03	0.003	19750	9.10	<.001	0.02 to 0.03
Trait ratings	0.05	0.004	19945	12.13	<.001	0.04 to 0.05
Word count	0.15	0.004	19954	40.81	<.001	0.15 to 0.16

Note. Linear mixed-effects modeling was implemented with a random intercept for the first participant in a pair, the second participant in a pair, and the celebrity. The observations for each pairwise analysis were doubled to allow each participant to be modeled as both the first and second participant in a pair for each observation (see methods section in main text). The resulting redundancy in the data was then accounted for by halving the estimated degrees of freedom and adjusting the p *value accordingly. All variables in the table above were modeled as pairwise similarity (as opposed to an Anna Karenina Model, e.g., mean scores, as was done for loneliness). Similarity in gender or race was modeled with a binary variable capturing whether two participants matched in their self-reported gender or race (1.0) or did not (0.0). Dissimilarity in ratings of liking, similarity, closeness, and familiarity as well as dissimilarity in word count were defined as the absolute difference between a pair. Dissimilarity in trait ratings was defined as the Euclidean distance between a pair's ratings in ten-dimensional space (trait dimensions: openness, conscientiousness, extraversion, agreeableness, neuroticism, warmth, competence, dominance, trustworthiness, and intelligence). Dissimilarity values were transformed to similarity values by* z *scoring them and flipping the sign. df = degrees of freedom. CI = confidence interval.*

Supplementary Table 3. *Multiple Predictor Model Predicting Pairwise Semantic Similarity for Descriptions of High-Consensus Celebrities.*

**Predictor**	**Beta**	**Standard Error**	**Satterthwaite Approximated *df***	***t* Value**	***p* Value**	**95% CI**
Mean Loneliness	-0.06	0.03	445	2.26	.01	-0.12 to -0.008
Gender	0.01	0.01	19678	2.47	.01	0.002 to 0.02
Race	0.02	0.01	20092	2.02	.04	-0.001 to 0.04
Age	0.03	0.004	19915	7.33	<.001	0.02 to 0.03
Liking	0.02	0.004	19816	6.13	<.001	0.01 to 0.03
Similarity	-0.01	0.004	19787	3.72	<.001	-0.02 to -0.01
Closeness	0.02	0.004	19861	5.58	<.001	0.01 to 0.03
Familiarity	0.02	0.003	19744	6.16	<.001	0.01 to 0.02
Trait ratings	0.03	0.004	19940	7.50	<.001	0.02 to 0.04
Word count	0.15	0.004	19954	40.83	<.001	0.14 to 0.16

Note. Linear mixed-effects modeling was implemented with a random intercept for the first participant in a pair, the second participant in a pair, and the celebrity. The observations for this pairwise analysis were doubled to allow each participant to be modeled as both the first and second participant in a pair for each observation (see methods section in main text). The resulting redundancy in the data was then accounted for by halving the estimated degrees of freedom and adjusting the p *value accordingly. All variables in the table above were modeled as pairwise similarity (as opposed to an Anna Karenina Model) with the exception of loneliness which was modeled as a pair's mean score. See Table S1 for a more detailed description of how the variables above were modeled. df = degrees of freedom. CI = confidence interval.*

Supplementary Table 4. *Single-Predictor Models Predicting Similarity to the Group-Consensus Semantic Representation for Descriptions of High-Consensus Celebrities.*

**Predictor**	**Beta**	**Standard Error**	**Satterthwaite Approximated *df***	***t* Value**	***p* Value**	**95% CI**
Gender	-0.06	0.09	447	0.60	.55	-0.25 to 0.13
Race	-0.25	0.11	445	2.28	.02	-0.45 to -0.03
Age	0.06	0.05	446	1.33	.18	-0.03 to 0.15
Liking	0.04	0.05	44	0.75	.46	-0.06 to 0.13
Similarity	-0.07	0.05	105	1.37	.17	-0.15 to 0.02
Closeness	-0.05	0.05	206	1.08	.28	-0.14 to 0.04
Familiarity	0.07	0.05	434	1.48	.14	-0.03 to 0.17
Trait ratings	0.17	0.05	405	3.62	<.001	0.08 to 0.26
Word count	0.29	0.05	447	6.41	< .001	0.19 to 0.38

Note. Linear mixed-effects modeling was implemented with a random intercept for celebrity. Gender and race were modeled as binary variables with 0.0 indicating a participant identified with the majority group in the sample (male or White, respectively) and 1.0 indicating a participant identified with one of the non-majority groups in the sample. Trait ratings refers to participants' similarity to the group-consensus perception of a celebrity's traits (see methods section in main text for details). df = degrees of freedom. CI = confidence interval.

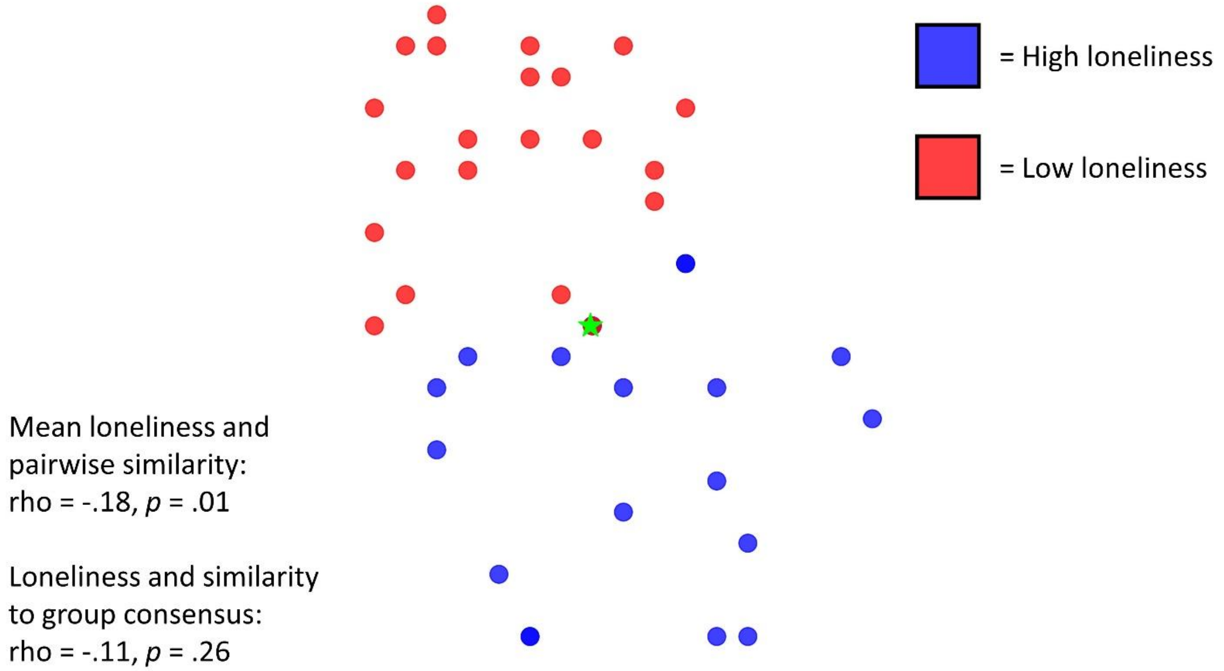

Supplementary Figure 1. X and Y coordinates were randomly generated for “low-loneliness” participants and “high-loneliness” participants, after which point the location of the “high-loneliness” cluster relative to the “low-loneliness” cluster was systematically changed to examine a wide array of different orientations. The figure above depicts an orientation in which there is a significant association between pairs’ mean loneliness scores and pairwise similarity in the randomly generated two-dimensional space, but no significant association between loneliness and similarity to the group-consensus. The green star denotes the coordinate with the greatest density of surrounding points. Note that though points are either blue (high loneliness) or red (low loneliness) based on a median split for the purpose of visualization, continuous loneliness scores matching those of the participants from Study 1A were used.

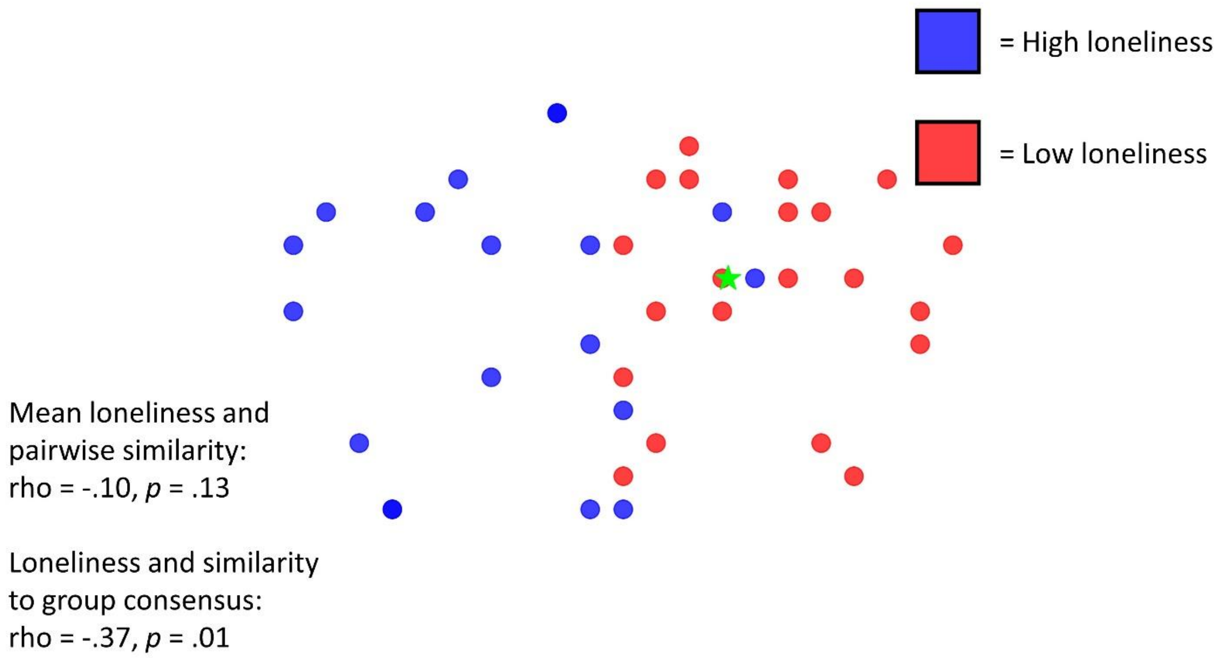

Supplementary Figure 2. X and Y coordinates were randomly generated for “low-loneliness” participants and “high-loneliness” participants, after which point the location of the “high-loneliness” cluster relative to the “low-loneliness” cluster was systematically changed to examine a wide array of different orientations. The figure above depicts an orientation in which there is no significant association between pairs’ mean loneliness scores and pairwise similarity in the randomly generated two-dimensional space, but a significant association between loneliness and similarity to the group-consensus. The green star denotes the coordinate with the greatest density of surrounding points. Note that though points are either blue (high loneliness) or red (low loneliness) based on a median split for the purpose of visualization, continuous loneliness scores matching those of the participants from Study 1A were used.

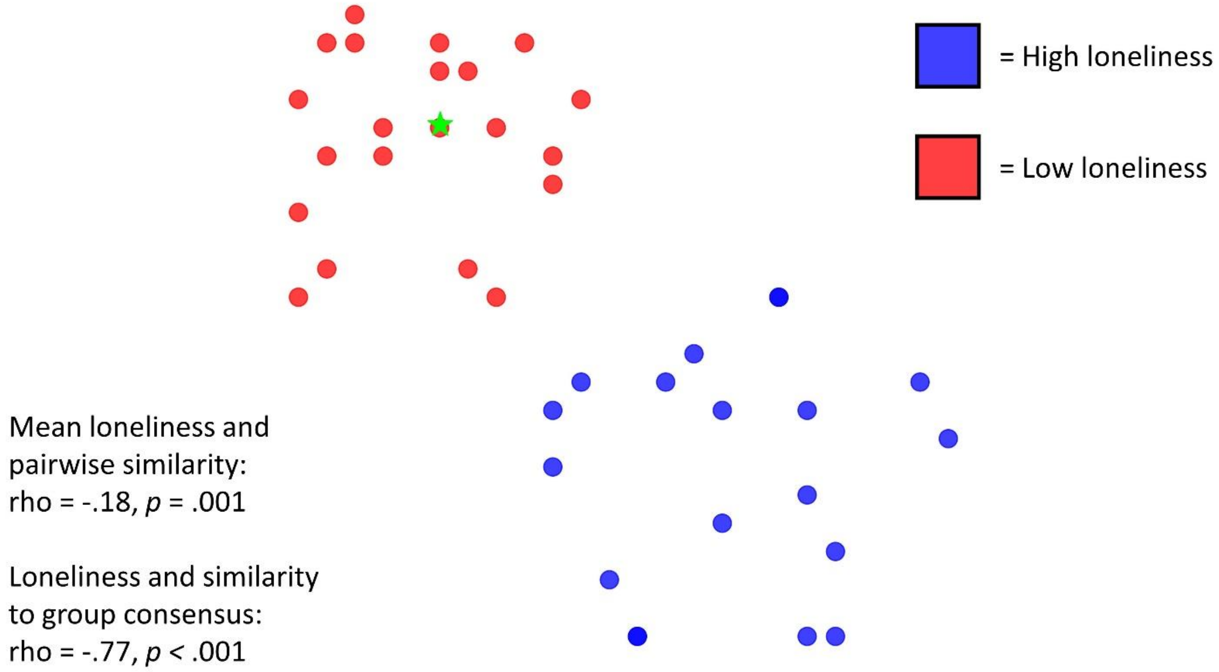

Supplementary Figure 3. X and Y coordinates were randomly generated for "low-loneliness" participants and "high-loneliness" participants, after which point the location of the "high-loneliness" cluster relative to the "low-loneliness" cluster was systematically changed to examine a wide array of different orientations. The figure above depicts an orientation in which there is a significant association both between pairs' mean loneliness scores and pairwise similarity in the randomly generated two-dimensional space and between loneliness and similarity to the group-consensus. The green star denotes the coordinate with the greatest density of surrounding points. Note that though points are either blue (high loneliness) or red (low loneliness) based on a median split for the purpose of visualization, continuous loneliness scores matching those of the participants from Study 1A were used.

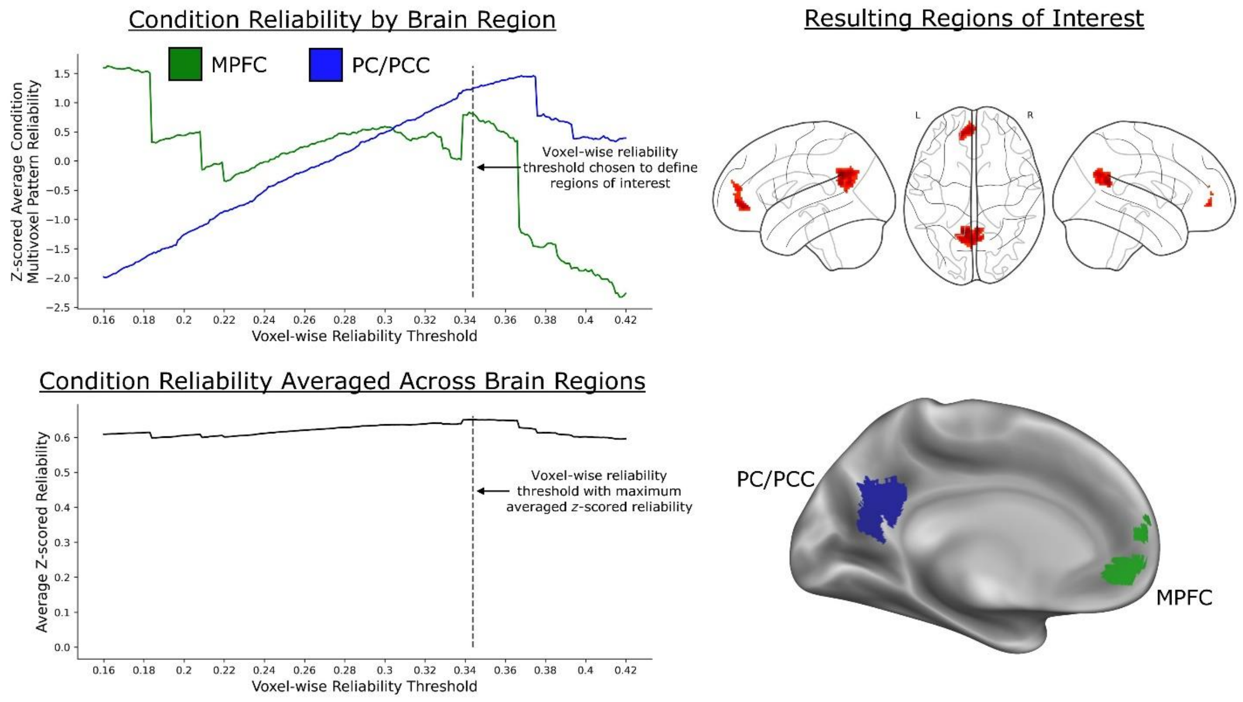

Supplementary Figure 4. Reliability-based voxel selection (RBVS) was implemented using the data from Study 1A (N = 40 participants) to define regions of interest (ROI) in the MPFC and PC/PCC, which past work shows reliably represent person knowledge. The x-axes display voxel-wise reliability thresholds. Voxel-wise reliability is computed by taking the vector of parameter estimates corresponding to the 16 conditions (i.e., target identities) in the study in one half of the data (i.e., odd runs) and correlating it with the corresponding vector in the other half of the data (i.e., even runs) at each voxel. Individual participant voxel-wise reliability maps were averaged into a single group map. The y-axis of the top left plot shows the z-scored average condition multivoxel pattern reliability for each ROI. The peak voxel (in terms of voxel-wise reliability) was identified within each brain region, and multivoxel pattern reliability for each condition was computed by vectorizing all voxels exceeding a given voxel-wise reliability threshold that formed a contiguous cluster with the peak voxel in a region and correlating this vector in one half of the data with the corresponding one in the other half. Multivoxel pattern reliability was averaged across all conditions for each participant. The average condition reliability across all participants was averaged into a single group-level value for all voxel-wise reliability thresholds ranging from .160 (the point at which the PC/PCC cluster was no longer connected to the visual cortex) to .420 (the point above which the MPFC ROI dropped below 33 voxels). Because the pattern of change in condition reliability was not consistent across the MPFC and PC/PCC, we *z* scored the condition reliability in each ROI across all thresholds examined and then averaged the *z*-scored reliability values for each ROI together (y-axis of bottom left plot). Vertical dotted lines show the threshold with the maximum averaged *z*-scored reliability/the threshold chosen to define the ROIs for the study (.344). The right side of the figure includes glass brain (top; made using Nilearn: https://nilearn.github.io/stable/index.html) and surface rendering (bottom; made using Connectome Workbench: https://www.humanconnectome.org/software/connectome-workbench) plots of the 208-voxel MPFC ROI and 525-voxel PC/PCC ROI. MPFC = medial prefrontal cortex. PC = precuneus. PCC = posterior cingulate cortex.

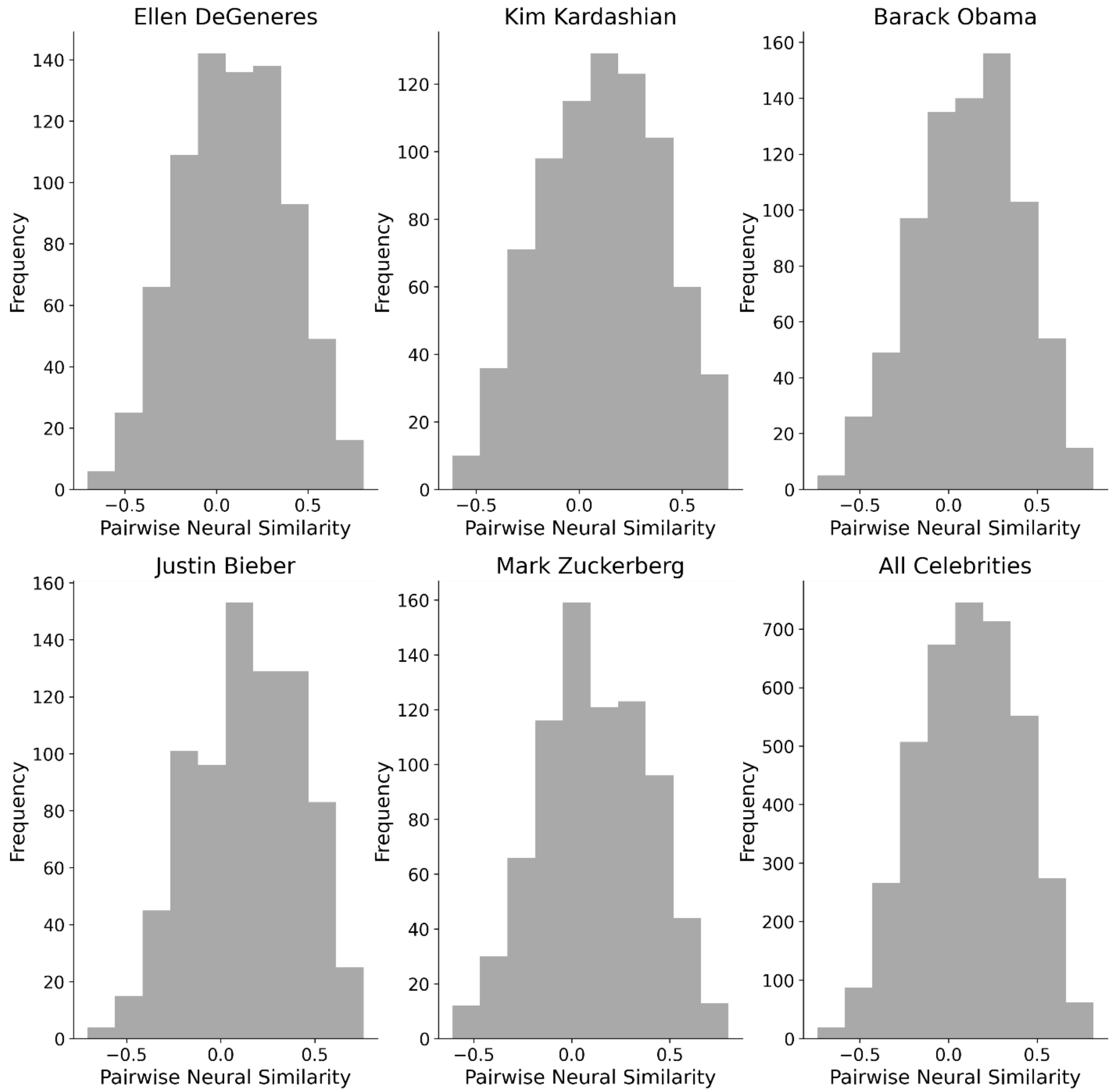

Supplementary Figure 5. Histograms showing the data distributions for the outcome variables for the main pairwise analyses in Study 1A. Pairwise neural similarity refers to the Pearson correlation between two participants' neural representations of a given celebrity in the medial prefrontal cortex region of interest.

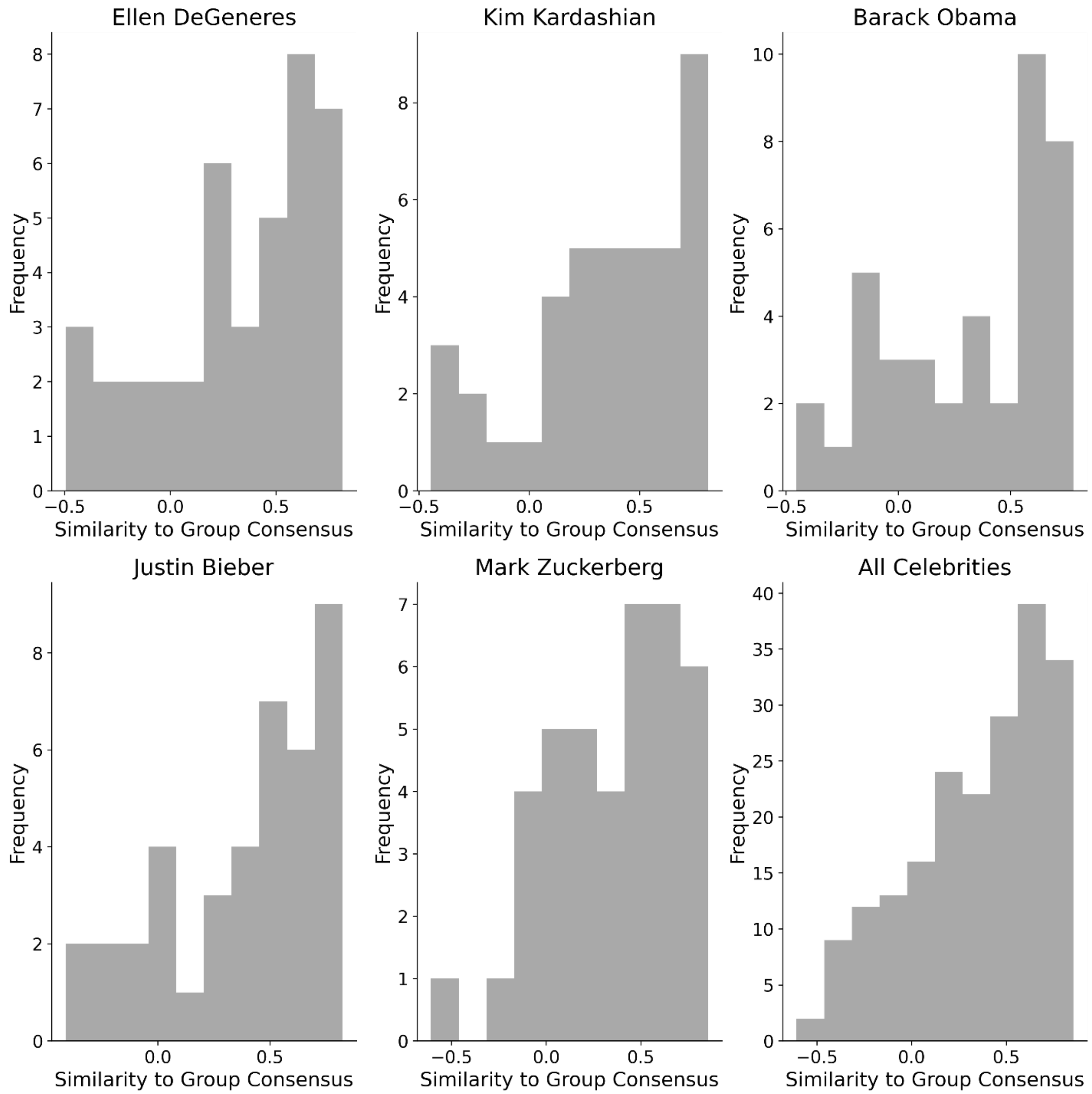

Supplementary Figure 6. Histograms showing the data distributions for the outcome variables for the main analyses examining similarity to the group-consensus neural representation of a given celebrity in Study 1A. Similarity to group consensus refers to the Pearson correlation between a participant's neural representation of a given celebrity and the group-consensus neural representation of that celebrity in the medial prefrontal cortex region of interest. See methods section of main manuscript for details regarding the computation of the group-consensus neural representation.

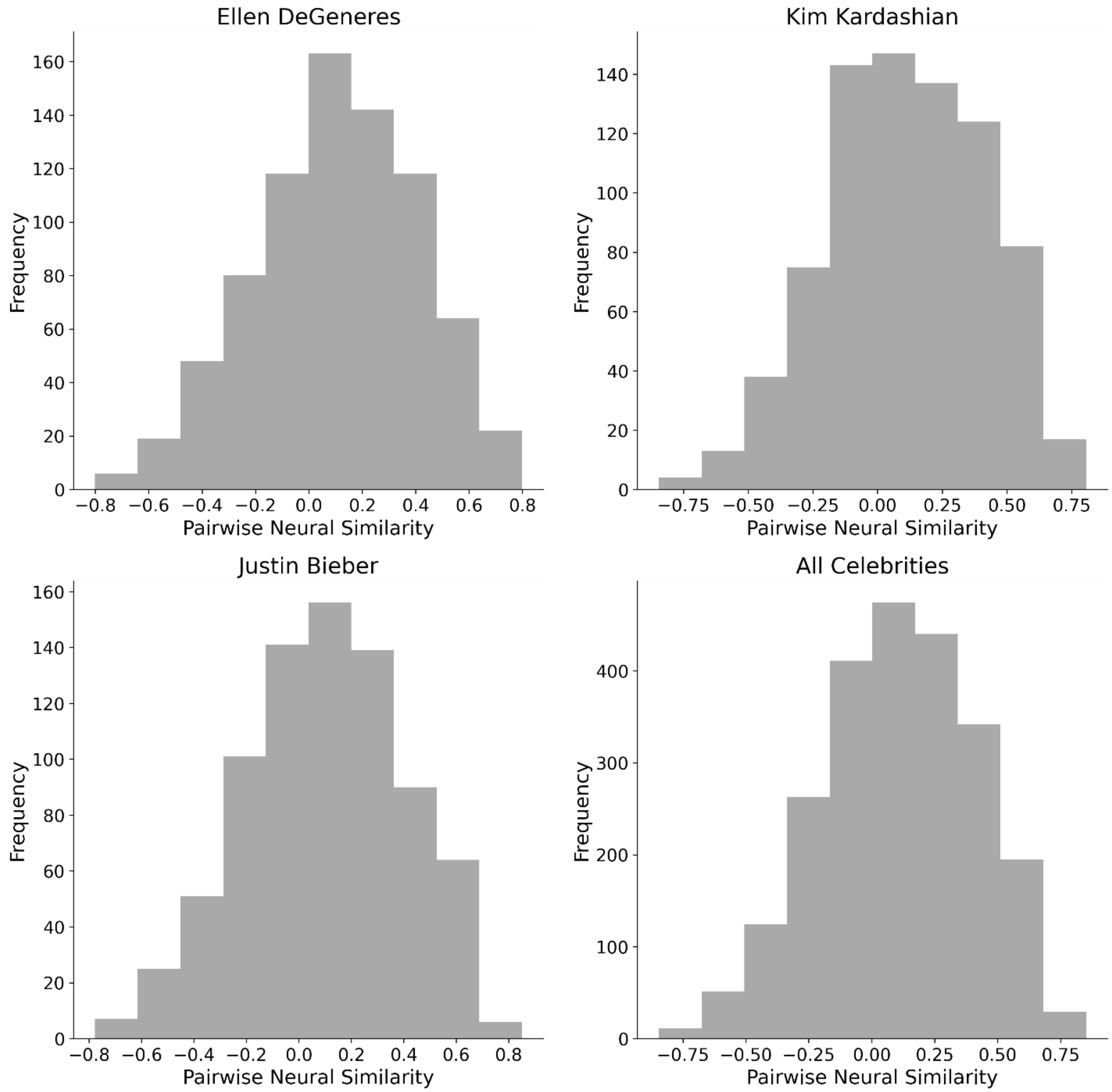

Supplementary Figure 7. Histograms showing the data distributions for the outcome variables for the main pairwise analyses in Study 1B. Pairwise neural similarity refers to the Pearson correlation between two participants' neural representations of a given celebrity in the medial prefrontal cortex region of interest.

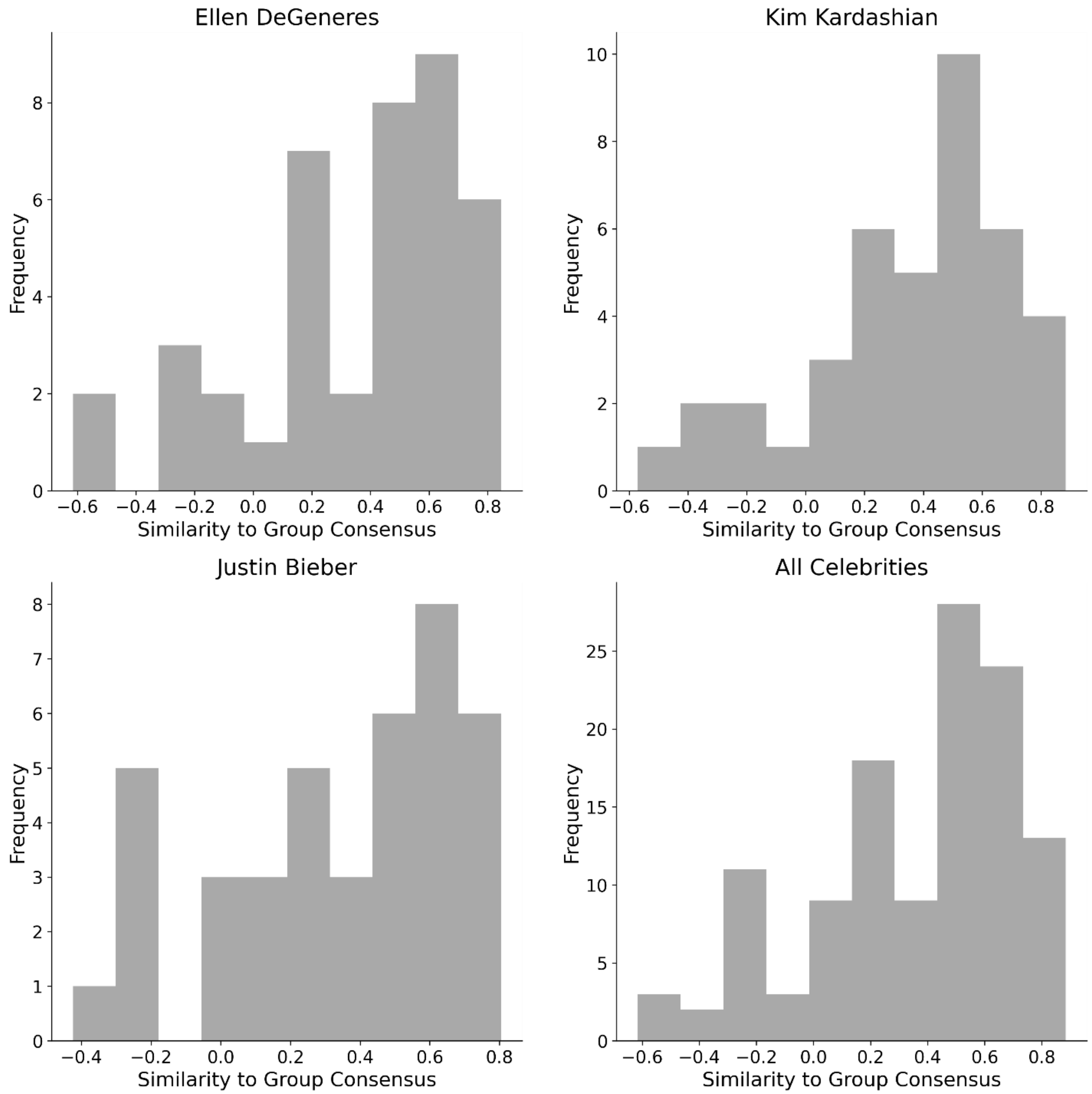

Supplementary Figure 8. Histograms showing the data distributions for the outcome variables for the main analyses examining similarity to the group-consensus neural representation of a given celebrity in Study 1B. Similarity to group consensus refers to the Pearson correlation between a participant's neural representation of a given celebrity and the group-consensus neural representation of that celebrity in the medial prefrontal cortex region of interest. See methods section of main manuscript for details regarding the computation of the group-consensus neural representation.

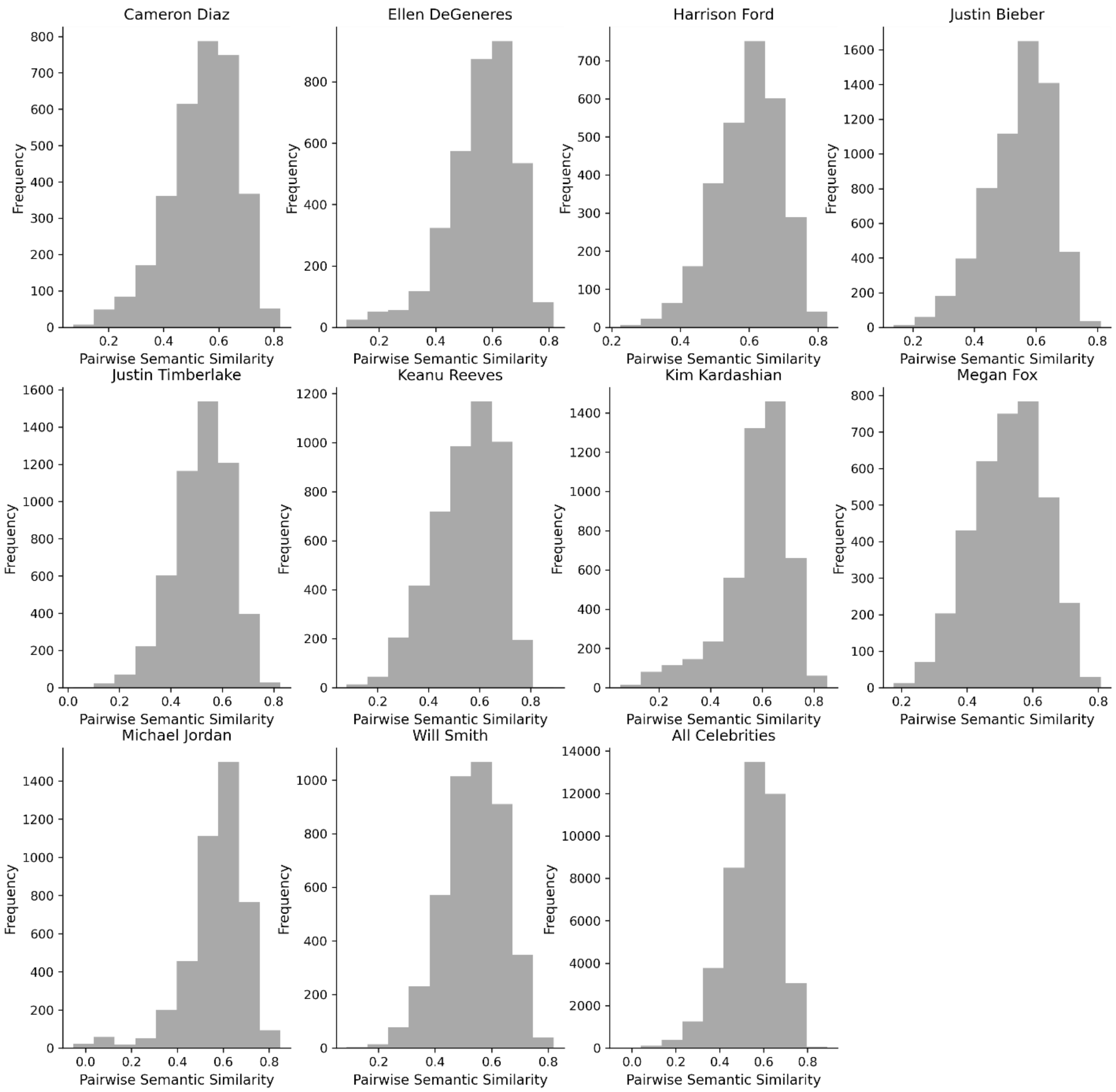

Supplementary Figure 9. Histograms showing the data distributions for the outcome variables for the main pairwise analyses in Study 2. Pairwise semantic similarity refers to the cosine similarity between two participants' semantic representations of a given celebrity derived using Google's Universal Sentence Encoder (see methods section of main manuscript for details).

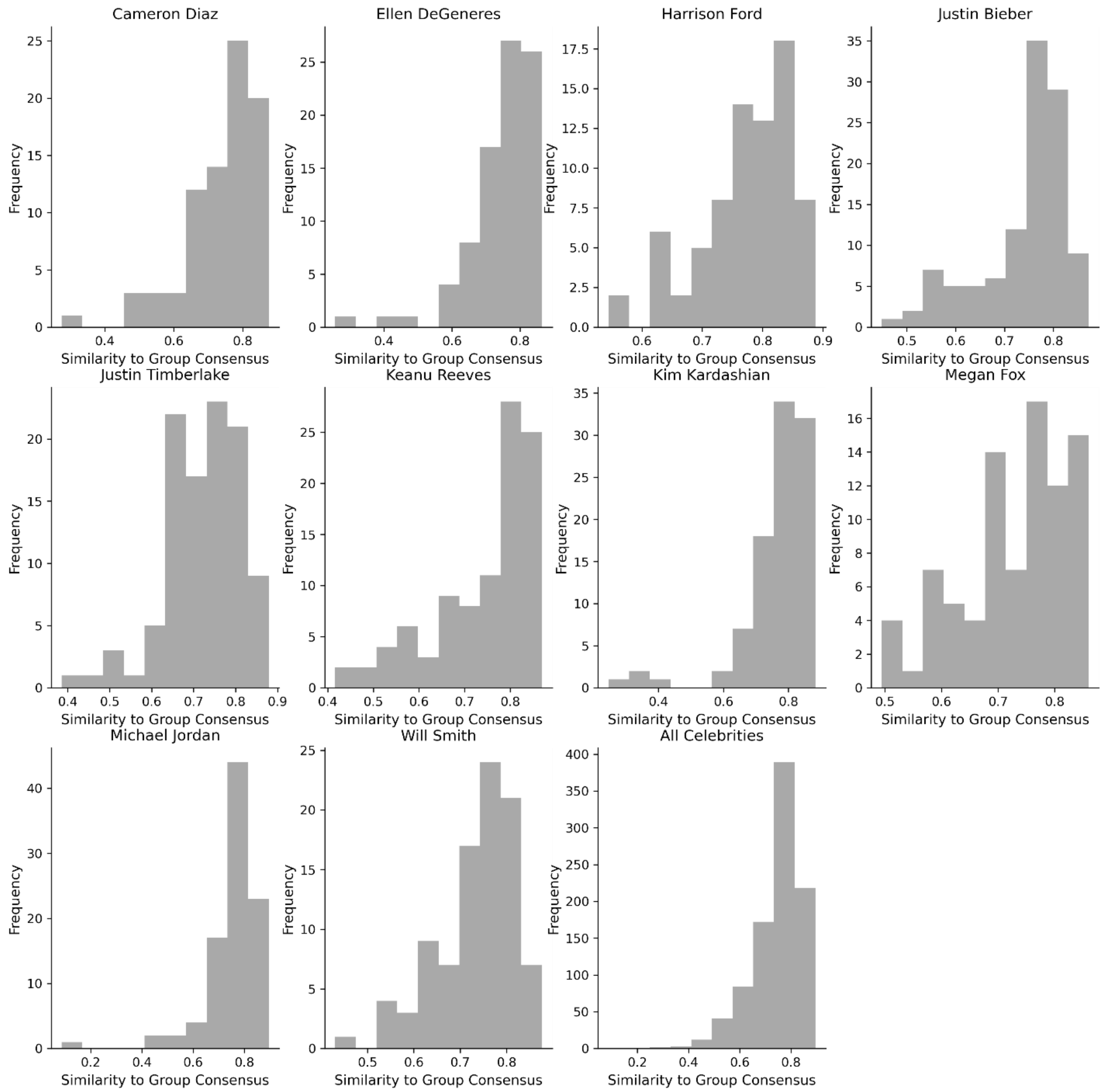

Supplementary Figure 10. Histograms showing the data distributions for the outcome variables for the main analyses examining similarity to the group-consensus semantic representation of a given celebrity in Study 2. Similarity to group consensus refers to the cosine similarity between a participant's semantic representation of a given celebrity derived using Google's Universal Sentence Encoder (see methods section of main manuscript for details) and the group-consensus semantic representation of that celebrity. See methods section of main manuscript for details regarding the computation of the group-consensus semantic representation.

Supplementary Methods

Simulations exploring the independence of results focused on pairwise similarity versus similarity to the group-consensus. We randomly generated X and Y coordinates (bound between zero and one and rounded to the first decimal point) for 21 “low-loneliness” participants (a median split on loneliness scores in Study 1A resulted in uneven group sizes due to some participants having the same loneliness scores), and 19 “high-loneliness” participants. The randomly generated coordinates for the “high-loneliness” participants were then multiplied by 1.5 to create greater distance between these points in line with the hypothesized pairwise effect (i.e., greater dissimilarity among lonelier pairs in their representations of celebrities). Next, we explored how the associations of interest changed as a function of where the “low-loneliness” and “high-loneliness” clusters were located relative to one another. This was achieved by “moving” the “high-loneliness” cluster around the “low-loneliness” cluster. We examined every possible combination of coordinates for the two clusters of points within the following constraints: the highest X or Y coordinate for “high-loneliness” cluster could not be lower than the lowest X or Y coordinate for the “low-loneliness” cluster, respectively, and the lowest X or Y coordinate for the “high-loneliness” cluster could not be higher than the highest X or Y coordinate for the “low-loneliness” cluster, respectively.

For every possible combination of coordinates for the two clusters of points, we tested for associations that mirrored those tested for in the actual study using the actual loneliness scores of the participants in Study 1A. First, we tested whether participants’ mean loneliness scores were associated with pairwise similarity (the inverse of the Euclidean distance between two points in the randomly generated two-dimensional space) by calculating the Spearman correlation between the two with statistical significance determined using a non-parametric permutation approach,

i.e., a Mantel test[2,3]. Next, we followed the same procedure to calculate a weighted-average group-consensus as is described in the main text (see materials and methods section), and calculated the Spearman correlation between loneliness scores and similarity to the group-consensus (the inverse of the Euclidean distance between a participant's coordinate and the "group-consensus" coordinate in the two-dimensional space).

Supplementary References

1. Tarhan, L., and Konkle, T. (2020). Reliability-based voxel selection. Neuroimage *207*, 116350.

2. Mantel, N. (1967). The detection of disease clustering and a generalized regression approach. Cancer Res. *27*, 209–220.

3. Finn, E.S., Glerean, E., Khojandi, A.Y., Nielson, D., Molfese, P.J., Handwerker, D.A., and Bandettini, P.A. (2020). Idiosynchrony: From shared responses to individual differences during naturalistic neuroimaging. Neuroimage *215*, 116828.
